# Group Cooperation without Group Selection: Modest Punishment Can Recruit Much Cooperation

**DOI:** 10.1371/journal.pone.0124561

**Published:** 2015-04-20

**Authors:** Max M. Krasnow, Andrew W. Delton, Leda Cosmides, John Tooby

**Affiliations:** 1 Department of Psychology, Harvard University, Cambridge, Massachusetts, United States of America; 2 Department of Political Science, College of Business, Center for Behavioral Political Economy, Stony Brook University, Stony Brook, New York, United States of America; 3 Center for Evolutionary Psychology, University of California Santa Barbara, Santa Barbara, California, United States of America; 4 Department of Psychological and Brain Sciences, University of California Santa Barbara, Santa Barbara, California, United States of America; 5 Department of Anthropology, University of California Santa Barbara, Santa Barbara, California, United States of America; Universidad Carlos III de Madrid, SPAIN

## Abstract

Humans everywhere cooperate in groups to achieve benefits not attainable by individuals. Individual effort is often not automatically tied to a proportionate share of group benefits. This decoupling allows for free-riding, a strategy that (absent countermeasures) outcompetes cooperation. Empirically and formally, punishment potentially solves the evolutionary puzzle of group cooperation. Nevertheless, standard analyses appear to show that punishment alone is insufficient, because second-order free riders (those who cooperate but do not punish) can be shown to outcompete punishers. Consequently, many have concluded that other processes, such as cultural or genetic group selection, are required. Here, we present a series of agent-based simulations that show that group cooperation sustained by punishment easily evolves by individual selection when you introduce into standard models more biologically plausible assumptions about the social ecology and psychology of ancestral humans. We relax three unrealistic assumptions of past models. First, past models assume all punishers must punish every act of free riding in their group. We instead allow punishment to be probabilistic, meaning punishers can evolve to only punish some free riders some of the time. This drastically lowers the cost of punishment as group size increases. Second, most models unrealistically do not allow punishment to recruit labor; punishment merely reduces the punished agent’s fitness. We instead realistically allow punished free riders to cooperate in the future to avoid punishment. Third, past models usually restrict agents to interact in a single group their entire lives. We instead introduce realistic social ecologies in which agents participate in multiple, partially overlapping groups. Because of this, punitive tendencies are more expressed and therefore more exposed to natural selection. These three moves toward greater model realism reveal that punishment and cooperation easily evolve by direct selection—even in sizeable groups.

## Introduction

Humans everywhere cooperate in groups. People band together to defend their village from raids. They build communal irrigation systems to water their crops. They collaborate in academic teams to publish scholarly papers. Indeed, our hunter-gatherer ancestors typically shared food widely to cover one another’s foraging shortfalls—a pattern believed to be characteristic of our lineage for hundreds of thousands if not millions of years [1]. In every known society past and present, people in groups made and make sacrifices to produce shared benefits. By acting in concert, humans achieve high-value outcomes that otherwise would have remained out of reach.

Human group cooperation comes in many forms. Sometimes participants automatically get a proportionate share of the benefits produced by group cooperation and sometimes non-participants are easily excluded from the group benefits [2,3]; understanding these types of cooperation is important. However, in this paper we focus on group cooperation that has the form of public goods. By definition, public goods can be exploited by free riders—people who have not contributed yet nonetheless take group benefits. The fact that humans regularly cooperate in public goods despite the threat of free riding [4,5] raises difficult theoretical questions about how natural selection could have favored its evolution and maintenance. Compare group cooperation in public goods (hereafter simply “group cooperation”) to individual foraging. Although the psychology of foraging is complex (e.g., [6,7,8]), it is easy to understand why it evolves: more or better food increases individual fitness and therefore selects for neural design features that improve foraging decisions and capacities. In contrast, group cooperation delinks expenditures from benefits, opening the group cooperation strategy to exploitation by free riding, a higher-payoff strategy that outcompetes it: Free riders, who receive a standard share of the collectively produced good while undercontributing to its production, will have higher fitness than group cooperators. This gives free riders a selective advantage that, if unchecked, will prevent the evolutionary maintenance or emergence of group cooperation. Indeed, economizing on effort when nothing is gained by exertion (the central principle of free-riding) is not inherently a hard to evolve nor difficult to implement strategy, but rather is likely to be the default design of organisms unless there are conditions where it is specifically selected against. Yet group cooperation does exist, and appears to be evolutionarily stable, so it follows that there must be a solution to the problem posed by free riders.

Debates about the evolution of group cooperation have turned on how the free rider problem is solved. One set of theories proposes that group cooperation evolves—and the free rider problem is solved—as the by-product of a psychology for learning arbitrary norms in tandem with cultural group selection: Some groups have norms that sustain cooperation, others do not, and cultural group selection tends to favor cooperative norms [9,10]. Another set of theories proposes that a psychology for group cooperation is a universal feature of human nature [11,12,2,13–16,3]. This universal and specialized psychology is what allows humans everywhere to effortlessly and easily cooperate in groups. On this view, moreover, the psychology of group cooperation exists because it brings individual or inclusive fitness benefits. Although it is harder to see than with foraging, group cooperation also creates direct benefits. On this latter view, the free rider problem can be solved without group selection.

Both the theory and evidence for cultural selection have been questioned [17–19], but it remains a widely accepted theory for the evolution of group cooperation. This is partly because the most prominent models of group cooperation were developed in this tradition. Cultural selection models, moreover, appear to explain gaps that other theories cannot. Chief among these gaps is how to solve the free rider problem without unduly handicapping cooperation. We suggest that these gaps are mostly illusory and have been created by failing to consider a few, very basic features of the real world. As we show in a simple model, once these real world considerations are taken into account, the free rider problem can be solved—and group cooperation can evolve—without cultural group selection. This solution depends on a new way of modeling the psychology of punishment.

## Solving the Free Rider Problem through Punishment

Researchers typically study two broad classes of potential solutions to the free rider problem, withdrawing from cooperation or punishing free riders (e.g., [20,21], for a different approach, see [22]). Withdrawal is problematic because it has devastating side-effects: Although withdrawal by cooperators does prevent exploitation by free riders, their withdrawal causes them to lose out on the benefits of cooperation. This is a steep cost to pay to solve the free rider problem. Punishment of free riders avoids the steep costs of withdrawal. By targeting free riders directly, punishment allows cooperators to prevent the free rider problem while still retaining the benefits of cooperation. Punishment, however, has its own drawbacks. It creates a *second-order free rider problem*: Cooperators who do not punish benefit at the expense of cooperators who do punish. Over time then, non-punishing cooperators will displace punishers. With punishers sufficiently diminished, non-punishers are once again vulnerable to free riders [20].

Punishment can have at least two functions. One function of punishment is *fitness reduction*: Punishment can directly reduce the relative fitness of free riders, closing the fitness gap between free riders and punishers (e.g., [23,24]). Another function is *labor recruitment*: Punishment can cause free riders to cooperate (e.g., [20,25]). Across many animal species, physical aggression or the threat of physical aggression is used to negotiate for improved standards of treatment [26]. The logic is simple: By demonstrating to others that there are costs to treating you poorly, you incentivize better future treatment toward yourself or your allies [27]. Anger in humans appears designed to fulfill precisely this role, including in cooperative relationships [28]. Thus, in the context of group cooperation, punishment may serve a labor recruitment function and cause free riders to become cooperators.

Boyd and Richerson [20] explored the potential of labor recruitment through punishment to stabilize group cooperation. In their model, punishers could induce free riders to cooperate. They found that when both cooperation and punishment are group beneficial but individually costly, both are selected out of the population. However, if the private cost of punishment yields a long-term profit to the punisher by inducing free riders to cooperate, then all three strategies can coexist at a mixed equilibrium. In this case of frequency dependent selection, rare punitive cooperators are able to cost-effectively recruit labor, giving them greater fitness than non-punitive cooperators. When punitive cooperators are common, however, non-punitive cooperators can (second-order) free ride on their efforts and are able to increase in frequency. Finally, when non-punitive cooperators increase in frequency, their willingness to be exploited favors the evolution of free riders. Thus, the population ends up in a mixed-equilibrium of punitive cooperators, non-punitive cooperators, and free riders. Because punitive and non-punitive cooperators both cooperate, and because many free riders are induced to cooperate, this result suggests substantial cooperation would be achieved at this equilibrium.

Since Boyd & Richerson [20], most models have studied fitness reduction, not labor recruitment [23,29–32]. In these models, punishers also direct punishment at free riders. However, it is not the recruitment of labor that allows punishers to prevail. Punishers prevail over free riders simply because punishment is *efficient*: It is more costly to be punished than to do the punishing. Thus, punishers have greater fitness than free riders, seemingly solving the free rider problem. It is not clear, however, that it is a solution to the *second-order* free rider problem. In fact, some theorists have concluded that, in models like these, processes of cultural selection are required to solve the second-order free rider problem (see [33]).

There are other problems with fitness reduction as typically modeled. In many models, free riders remain free riders, never responding to punishment (e.g., [30,34]). Such an assumption is problematic, because it does not seem to match actual human behavior, the behavior of other animals [26], or minimum agent rationality. In other models, fitness reduction is combined with social learning. In these models, a free rider whose fitness is reduced below cooperators’ fitness might switch strategies and become a cooperator [23,24]. Unfortunately, this is not a general solution: As is widely acknowledged in models of cultural learning, this process does not uniquely pick out cooperative equilibria. In models like these, punishment can stabilize anything, not just cooperative behaviors. Further processes of cultural selection are then required to explain why the cooperative equilibria predominate [10,35].

Given that Boyd & Richerson [20] found an equilibrium where a substantial amount of cooperation was sustained, why have theorists focused so much attention on fitness reduction and cultural selection? We are not entirely sure, but one possibility is that Boyd & Richerson were themselves skeptical of their result. Because of their skepticism, they developed another model wherein not only did punishers punish free riders, punishers also punished non-punishing cooperators (a questionable assumption about real-world humans, see [36]). The result, similar to models combining fitness reduction and social learning, is that anything and everything—not just cooperation—could be stabilized by punishment. This conclusion led to the proliferation of cultural selection models designed to explain how cooperative equilibria are picked out from among the many other possible equilibria [10,33,35]. We think such a conclusion is premature. Our goal with this paper is to reevaluate the possibility for labor recruitment through punishment to explain group cooperation and solve the free rider problem—and do so using more biologically realistic assumptions.

## The Present Research

We suggest that at least some of these issues could be resolved by returning punishment to its ecologically obvious role of recruiting labor: By inducing free riders to cooperate, punishment is individually beneficial. In order to explore this possibility, we introduced several straightforward features into the model. First, we made the cost of being punished exceed the net cost of cooperating (the *net* cost of cooperating is the cost of cooperation less the personal gain generated). If we had not built in this minimal feature, then being exposed to punishment would not decrease the payoffs to free-riding sufficiently to either select against free-riding or to induce free-riders to shift their behavior to cooperation—i.e., to incentivize their recruitment into the ranks of the productive. Their best choice would still be free-riding. The second feature we introduced is that agents recognize those who have punished them, so they have the potential to evolve to respond differentially to those who have punished them in the past.

We study a model that has four advantages over past models of labor recruitment. First, in past work, punitive strategies were modeled as discrete types: punishers punished every free rider every time and every punisher in the group punished every free rider in the group. While the first punisher in a group can induce every free rider to cooperate, there is no marginal gain from additional punishers. Because of this, as group size increases, more and more punishment is wasted on free riders who have already been induced to cooperate. Although assuming discrete types simplifies analytic investigation (a nontrivial benefit), real world behavior is more textured. Real-world decision systems evolved to operate in situations that involved graded continua, and often the best response involves gradations. Moreover, laboratory studies, for example, often find considerable between-subject and within-subject variability in behaviors like costly cooperation and punishment (e.g., [37,38]). In contrast to discrete types, we therefore model strategies as continuous: Although all agents in our model can punish free riders, whether they actually do depends on an internal psychological variable that encodes the probability of punishing. Critically, the value of this variable is evolvable. Thus, selection can move this variable to 0% (never punish anyone), to 100% (always punish every free rider), or to any value in between (meaning some but not all encountered free riders will be punished). In contrast to the case of discrete types, with probabilistic punishment the costs of punishment are spread among the punitive tendencies of the group members. This allows punishment to evolve without punishers being forced to punish every free rider every time.

Continuously varying dispositions to punish can also solve a related problem: Previous models have been used to support the claim that that even if group cooperation could evolve without cultural processes, this would only be true when groups are very small. In contrast, in our model, the costs of punishment do not have to increase as group size increases, because agents are not forced by model assumptions to punish every free rider every time. This novel feature allows the evolution of group cooperation across a much larger range of group sizes (see [14], for another approach to using continuous strategies to allow the evolution of large groups).

Second, we modeled a less arbitrarily restrictive (and more natural) psychology of cooperation, again using continuous probabilities. We removed the restriction that the default propensity to cooperate had to be binary and discrete (e.g., cooperate/not cooperate). Instead, agents consult a continuous psychological variable to determine the probability that they cooperate. As with willingness to punish, selection can move the probability of default cooperation anywhere from 0%, to 100%, to anywhere in between. We call it “default” cooperation because it encodes the probability of cooperation when an agent has not been induced to cooperate by punishment. This feature allows us to test how much default cooperation will evolve in the presence of punishment.

The variables specifying the probability of default cooperation and the probability of punishing are completely independent of each other and can evolve separately. This allows for the evolution of agents in all four quadrants of strategy space: i.e., willing to both punish and cooperate; unwilling to either punish or cooperate; willing to cooperate but not punish; and willing to punish but not cooperate. The only possibility we do not allow is that agents cannot punish cooperators. Punishment of cooperators can alter evolutionary dynamics [39], but modeling it is beyond the scope of the present paper and awaits future investigation.

Third, we assume that when a punisher induces a free rider to cooperate, that happens through individual recognition. Consider an agent F who free rode and was then punished by agent P. In the future, agent F will cooperate in the presence of agent P because of the past punishment. Importantly, agent F will *not* cooperate due to agent P’s punishment if P is not present; agent P’s punishment only recruits F’s labor when P is present. (Of course, even when P is not around, F could cooperate for other reasons, such as F’s default propensity to cooperate or the presence of others who have previously punished F.) In general, this means that an agent who has been punished for free riding will only cooperate due to punishment in the presence of another agent who has previously punished them.

Note that in our model there is no third-party reputation for punishment: Agents only learn that someone else is a punisher if they are directly punished by the punisher; they cannot learn that another agent is willing to punish by observing them punish someone else. We limited our model in this way because we did not want to bias the model towards the evolution of punishment and cooperation. Other work suggests that labor recruited by a reputation for punishment can indeed be stable in large groups, but they only considered a social ecology where reputation immediately spreads throughout the population [40]. Whether or not this assumption is correct, we did not want our model to hinge on it. By omitting reputation effects, it will be all the more striking if our model still shows that punishment and cooperation can evolve by individual selection.

Fourth, researchers typically model group cooperation by assuming a social ecology of fixed groups, where agents interact in a single group for their entire life. Although agents might go through multiple rounds of choosing to cooperate and choosing to punish, for each agent this happens within a single, permanent group. Thus, punitive tendencies that could recruit labor are only briefly exposed to natural selection in the first round of interaction; after the first round, anyone who was a free rider has been punished and is now cooperating. However, real world group cooperation is often temporary and task-specific (e.g., hunting or raiding parties), and forager band structure is frequently fluid, often involving temporary fissions, transfers, and fusions. Real world groups might end when their task is accomplished or their memberships might change over time, a feature recognized by evolved human psychology [41–43]. In our everyday lives we engage in many overlapping, shifting, and temporary cooperative enterprises. In such a *mixing ecology*, punitive tendencies will be continually re-exposed to natural selection, and if punishment returns a net benefit, it should correspondingly evolve to a higher level. Like the case with discrete types, the fixed groups assumption simplifies formal analysis. Because natural selection acts on the average fitness effects of genes, not the individuals in which they reside, this is generally a safe assumption. But we suspect that in this instance the assumption under-exposes punitive tendencies to natural selection and thus underestimates how much punishment can evolve, especially in agent-based simulations.

In short, realistic ecological assumptions suggest that punishment can create direct benefits by recruiting labor—by inducing free riders to cooperate. Moreover, these direct benefits may outweigh the benefits that punishment confers on non-punishing cooperators, minimizing or avoiding the second-order free-rider problem. Thus, punishment of free riders and the maintenance of group cooperation could emerge merely by individual-level selection without requiring group selection or cultural evolutionary processes and could even do so for large groups.

## Methods

The following simulations were conducted to test the hypotheses that quantitative traits of punishment and cooperation and mixing ecologies would help mitigate the second-order free-rider problem and allow the evolution of punishment and group cooperation (simulation software written in Java and available upon request). For each simulation run, a meta-population of N = {250, 500, 750, 1000, 1250} agents was randomly sorted into 5 populations of size N/5. Within each population agents were randomly sorted into 10 groups of size g = {5, 10, 15, 20, 25} which then engaged in a cooperative interaction. (Note that N and g are not independent; there are always 10 groups within a subpopulation, so once g is fixed, then N is fixed.) The long-run gains from cooperation were jointly determined by two parameters: One parameter, w, determined how many rounds of cooperation agents engaged in; the other, b, determined how much benefit was at stake in a given round of cooperation. The number of generations was fixed at 10,000. Within a generation, agents engaged in one or more rounds of a cooperative interaction. We ran 20 simulation runs for every possible combination of the within-round benefits of cooperation (b), the length of interactions (w), the group size for cooperative interactions (g), and the dichotomous parameter of social ecology (fixed groups versus mixing). For data analysis, from each simulation run we tabulated the average values of *Punish-Free Riding*_*Probability*_ and *Default-Cooperation*_*Probability*_ for the final 500 generations (data included in SI).

### The structure of the cooperative interactions

Every generation engaged in at least one round of a cooperative interaction. Cooperative interactions take place within the groups of size g. Each round had two phases, a cooperation phase and a punishment phase. In the cooperation phase each agent chooses to either cooperate or free ride. An agent who free rides pays no cost. An agent who cooperates pays a cost of 1 to generate a benefit b = (.2, .4, .6, .8, 1) for each group member; the size of b varied between simulations but was fixed within simulations. If b = .6 and every member of a ten person group cooperates then each member earns 5 (= .6 *10–1). Free riding, absent punishment, earns more: If one member now free rides but the others continue cooperating, the free rider earns 5.4 (= .6 * 9) whereas the cooperators each earn only 4.4 (= .6 * 9–1). There are also a few parameter combinations where free riding and cooperation have identical payoffs (for instance, when b = 1, the payoffs to the two choices are always identical). In general, however, everyone is better off as more group members cooperate, but it is individually payoff-maximizing to unilaterally free ride.

In the punishment phase, each agent chooses, independently for each free rider, whether to punish that free rider. All agents make these decisions, including agents who themselves free rode. In all simulations, punishers pay a cost of 1 to impose a cost of .9 on the free rider—in other words, doling out punishment is costlier than being punished. These values were chosen so that punishment is (slightly) ineffective, ruling out spite as an alternative selection pressure favoring punishment and making the model an even stricter test of the labor recruitment hypotheses.

Agents were endowed with two evolvable variables (genes) they used to generate behavior. The first variable—*Default-Cooperation*_*Probability*_—regulated the probability the agent cooperated by default, that is, without being induced to cooperate by current group members. The second variable—*Punish-Free Riding*_*Probability*_—regulated the probability the agent punished free riders. Each agent’s psychology contained a memory which recorded the identity of agents that had previously punished them. Given these variables and their memories, agents acted according to the following decision rules:

1. In the cooperation phase of the interaction, agents check if any other members of their group have previously punished them. If so, agents cooperate. Otherwise, agents cooperate with probability *Default-Cooperation*_*Probability*_.
2. In the punishment phase, agents evaluate each free rider and punish them with probability *Punish-Free Riding*_*Probability*_. This process is repeated independently for each free rider; punishing one free rider does not guarantee an agent will punish another.
3. Agents add the identity of anyone who has punished them to their memory. Note that agents only remember who punished them personally; they do not remember punishers who only punished others. Although this is not necessarily realistic, it works against our hypothesis by minimizing the labor that can be recruited by a single act of punishment.

Note that all agents have identical psychologies: They all follow the same decision rules and have the same procedure for updating their memory. Thus, there are not discrete types such as free rider, punisher, or cooperator, each with their own unique psychology. What does vary between agents are quantitative, evolvable parameters that determine how likely an agent is to punish or cooperate by default. We also note that while punishment is probabilistic, agents respond to punishment as if it signaled that any future free-riding would be punished. There are more cognitively sophisticated inferences that could be made, but we leave exploring them to future work. Though simple, this decision-rule captures the main dynamic of labor recruitment.

Our hypothesis is that it is the benefits achieved by recruiting labor that drives the evolution of punishment in group cooperation. To ensure that it is labor recruitment specifically that drives cooperation, not the act of punishment itself, we duplicated all simulations with one change: Agents were not allowed to remember that anyone had ever punished them. With no memory, agents cannot be induced to cooperate by the presence of a punisher. Thus, labor recruitment was impossible. Because punishment is ineffective—costing more to dole out than to receive—it is unlikely that punishment in the absence of labor recruitment could allow cooperation to evolve. Given the centrality of labor recruitment to our hypothesis, however, we wanted to actively check this assumption.

### Ecologies

In the first round of every generation, agents were randomly sorted into groups of size g within their population. In the fixed groups ecology, every round of cooperative interaction was with this same group. In the mixing ecology, the population was randomly sorted into new groups every round. New groups may or may not contain members that agents have interacted with before. Note that agents in the mixing ecology were only randomly grouped with members of their population, not with members of the larger meta-population.

After a guaranteed first round of interaction, they probabilistically moved onto to a second round according to the probability *w*. If the second round occurred, they moved onto the third round with probability *w*. If the third round occurred, they moved onto the fourth round with probability *w* and so on until cooperation probabilistically ended. For the fixed groups ecology, *w* had the values of .94, .95, .96, .97, .98, or.99; for the mixing ecology, *w* had the values of .994, .995, .996, .997, .998, or .999. These probabilities ensure that, independent of ecology type, the approximate expected number of future interactions between two individuals—and thus the potential labor to be recruited—was 16, 20, 25, 33, 50, and 100 interactions (calculated as$\frac{p}{1-w}$, where $p=\frac{group\;size}{population\;size}$ in the mixing ecologies, and p = 1 in the fixed groups ecology).

### Reproduction

When a generation terminates, the current generation reproduces and is completely replaced by the new generation. The new generation forms a new meta-population which is randomly sorted into new populations. This means that although fitness accrual happens based only on events within an agent’s population, reproduction happens at the level of the larger meta-population. In other words, agents’ children are not necessarily in the same population as them. The new meta-population is created using a standard method of modeling reproduction: For each member of the new generation, the probability an agent from the previous generation is their parent is the potential parent’s relative fitness. The more fit a member of the previous generation is, the more likely they are to be the parent.

When agents reproduce, their genes—the probability of default cooperation and the probability of punishing—are reproduced subject to mutation. Independently for each gene there is a 5% probability it will be mutated. If it is mutated, its value is changed by adding a random draw from a normal distribution with a mean of zero and a standard deviation of .05. Given that the genes represent probabilities, however, mutation cannot move the genes outside of the range 0 to 1. Although a mutation rate of 5% is larger than is typical for discrete strategies, it is appropriate for continuous strategies; with these parameters the genetic values of daughter generations differ through mutation alone by an average of 0.19%. To ensure some variability at the beginning of each simulation run, for each run the meta-population was initialized by setting the value of each agent’s genes to 0.1 and subjecting them to a 100% mutation rate.

Note that the values of each agent’s genes are independent of each other; all combinations of genetic values are possible. This means that second-order free riding is possible: In principle, selection can build agents very willing to cooperate but unwilling to punish. Thus, the model makes no assumptions whatsoever about punishment and cooperation being linked.

## Results

### Does punishment evolve in this new model?

**Fig 1:**
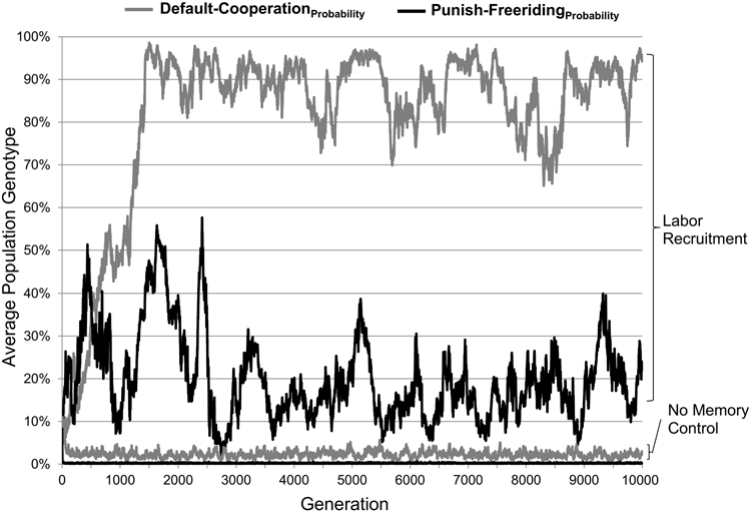
Sample evolutionary dynamics. This figure plots the average level of *Default-Cooperation*_*Probability*_ and *Punish-Freeriding*_*Probability*_ over generations from one representative simulation run from the original simulation and from the no-memory control. For these runs group size = 5, b = 0.8, expected encounters = 50, and it was a mixing ecology.

Yes. To show an example of the time course of the evolution of punishment, Fig 1 plots a sample of the evolutionary dynamics for the mixed ecology when b = .8, g = 5, and the expected number of repeat encounters was 50. In this run, the probability of punishing free riders increases beyond its initial value of .1, doubling to about .2: Agents will punish a free rider about 20% of the time. This implies that there is a 59% probability that at least one of the other four group members will punish the 5^th^ member who free rides (calculated as 1−(1−.2)^4^). So although a particular agent will usually not punish, in the group as a whole it is likely that someone will. Thus, the costs of punishment are spread throughout the group, easing the evolution of punishment.

**Fig 2:**
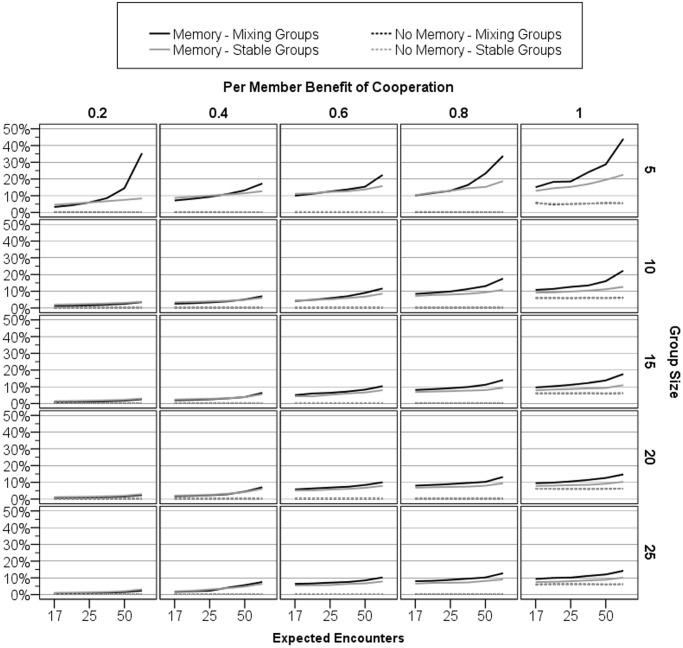
Final average values of *Punish-Freeriding*_*Probability*_. This figure plots the average willingness to punish of the last 500 generations of each simulation.

The evolution of punishment occurs broadly; Fig 2 plots the final levels of the probability of punishing free riding across the full parameter space we tested. When there are sufficient gains to cooperation—when the within-round benefits are large or when agents are likely to meet again—agents evolve to be willing to punish. This is further confirmed by a general linear model (GLM) analysis (Table 1) showing that the probability of punishing free riders increases with greater within-round benefits (F(4,5700) = 7592.62, p<.001, η^2^ = 0.35) or the greater expected number of encounters (F(5,5700) = 2038.84, p<.001, η^2^ = 0.12). (See Table 2. for similar model for the probability of default cooperation.)

**Table 1:** GLM of *Punish-Freeriding*_*Probability*_.

Source	SS	df	MS	F	Sig.	η ^2^
Intercept	37.737	1	37.737	159722.181	<.001	
Per Member Benefit	7.176	4	1.794	7592.619	<.001	.345
Expected Encounters	2.409	5	.482	2038.824	<.001	.116
Group Size	5.739	4	1.435	6072.057	<.001	.276
Ecology	.482	1	.482	2040.374	<.001	.023
Benefit * Encounters	.105	20	.005	22.318	<.001	.005
Benefit * Group Size	.222	16	.014	58.810	<.001	.011
Benefit * Ecology	.323	4	.081	341.768	<.001	.016
Encounters * Group Size	1.040	20	.052	219.985	<.001	.050
Encounters * Ecology	.423	5	.085	358.212	<.001	.020
Group Size * Ecology	.165	4	.041	174.805	<.001	.008
Benefit * Encounters * Group Size	.378	80	.005	20.020	<.001	.018
Benefit * Encounters * Ecology	.068	20	.003	14.459	<.001	.003
Benefit * Group Size * Ecology	.145	16	.009	38.246	<.001	.007
Encounters * Group Size * Ecology	.513	20	.026	108.524	<.001	.025
Benefit * Encounters * Group Size * Ecology	.240	80	.003	12.675	<.001	.012
Error	1.347	5700	.000			
Total	58.511	6000				
Corrected Total	20.774	5999				

**Table 2:** GLM of *Default-Cooperation*_*Probability*_.

Source	SS	df	MS	F	Sig.	η ^2^
Intercept	1426.508	1	1426.508	358807.623	<.001	
Per Member Benefit	505.860	4	126.465	31809.572	<.001	.795
Expected Encounters	4.669	5	.934	234.855	<.001	.007
Group Size	20.445	4	5.111	1285.594	<.001	.032
Ecology	.338	1	.338	85.133	<.001	.001
Benefit * Encounters	24.115	20	1.206	303.279	<.001	.038
Benefit * Group Size	33.461	16	2.091	526.031	<.001	.053
Benefit * Ecology	4.274	4	1.069	268.770	<.001	.007
Encounters * Group Size	.496	20	.025	6.238	<.001	.001
Encounters * Ecology	.866	5	.173	43.543	<.001	.001
Group Size * Ecology	.104	4	.026	6.532	<.001	.000
Benefit * Encounters * Group Size	15.603	80	.195	49.058	<.001	.025
Benefit * Encounters * Ecology	1.271	20	.064	15.979	<.001	.002
Benefit * Group Size * Ecology	.300	16	.019	4.724	<.001	.000
Encounters * Group Size * Ecology	.364	20	.018	4.577	<.001	.001
Benefit * Encounters * Group Size * Ecology	1.868	80	.023	5.874	<.001	.003
Error	22.661	5700	.004			
Total	2063.204	6000				
Corrected Total	636.696	5999				

### Does punishment select for a default tendency to cooperate?

**Fig 3:**
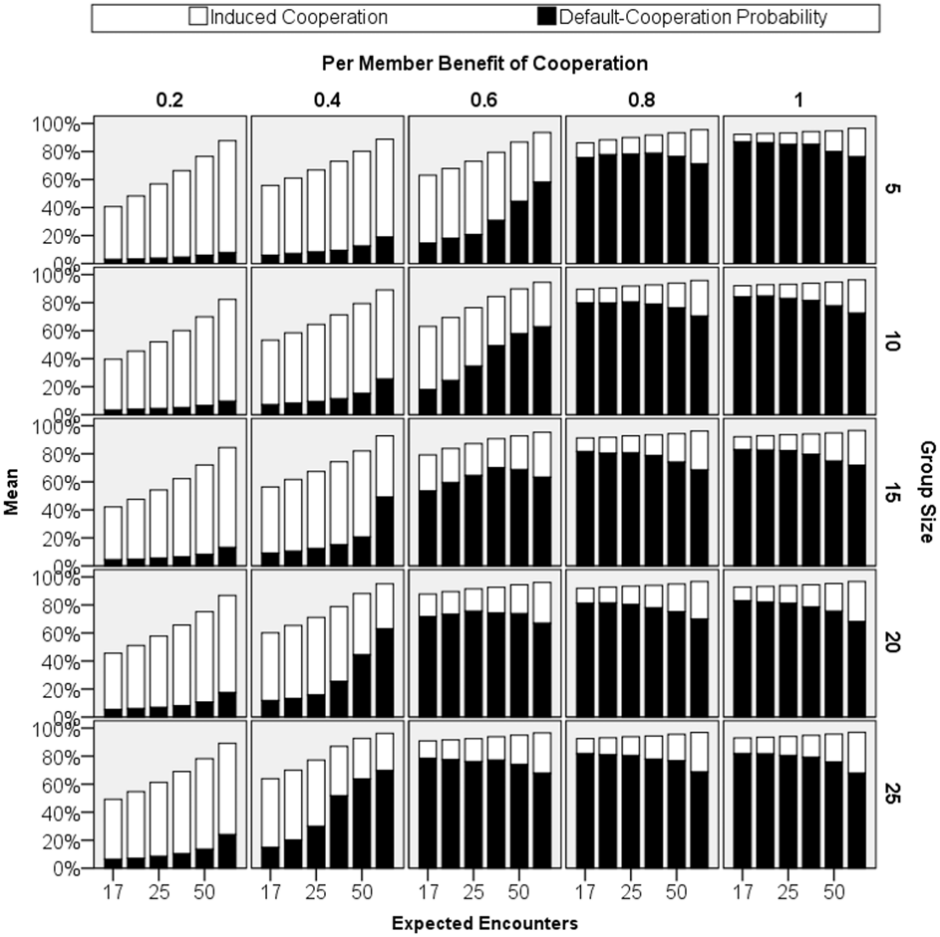
Final averaged cooperation rate (genetic & induced). This figure plots the average cooperation rate of the final round of the last 500 generations of each simulation. The full height of each bar indicates the cooperation rate. The cooperation rate is decomposed into cooperation induced by punishment (white) and cooperation given by the gene *Default-Cooperation*_*Probability*_ (black).

Yes. In the example of Fig 1, the modest amount of punishment that evolves is enough to support much default cooperation: The probability that an agent cooperates—without being induced by a punisher—quickly rises to around 90%. This means that 60% of the time everyone in the group cooperates even without being induced by punishment. Default cooperation also broadly evolves in; Fig 3 shows the final levels of the probability of default cooperation across all the primary simulation runs (black bars). When there are sufficient gains from cooperation, default cooperation evolves, sometimes substantially so.

Greater default cooperation is associated with greater willingness to punish: Across simulations, these two values were correlated (r(5998) = .51, p<.001). This was only true, however, at the between-simulation level; within populations there was no correlation between default cooperation and willingness to punish (based on testing the mean within-population correlation against zero; M = .000, SD = 0.023, t(5999) = 0.82, *ns*.). The fact that cooperation and punishment are not correlated at an individual level is of additional interest given that many analytic models of group cooperation and punishment explicitly assume that the willingness to cooperate and to punish are linked (e.g., [20]) and this linkage forms the basis of strong reciprocity proposals [9,44,45]. This result challenges this assumption.

### How much cooperation does our model support?

The probability of default cooperation does not measure all cooperation; it only captures cooperation that occurs *without* inducement through punishment. When we account for cooperation induced by punishment, the total amount of cooperation is substantial. Fig 3 graphs in white bars the observed cooperation rates. If cooperation rates were determined solely by the probability of default cooperation, these bars should not be visible; they would match exactly the black bars graphing default cooperation. Instead, the graph shows that actual cooperation rates can be substantial even in cases where the probability of default cooperation is not itself high. Sometime the two sources of cooperation combine to produce cooperation rates upwards of 95%.

Modest punishment supports much cooperation: Consistent with our theory, probabilistic punishment allows the costs of punishment to be spread across the group. It does not take much punishment to induce a lot of cooperation; this can be seen especially when groups are large. For instance, when group size is 20 and within-round benefits of cooperation are .8, agents are willing to punish only about 10% of free riders (Fig 2). However, with so many group members, there is usually at least someone who has previously punished a given free rider. This causes the total cooperation rate to be 90% or more (Fig 3).

### Is it labor recruitment, and not spite, that allowed the evolution of punishment and cooperation?

Yes. This can be seen by comparing the baseline simulation set (where labor recruitment was possible) to the no-memory condition (where labor recruitment was impossible). In the example graphed in Fig 1, the probability of punishing free riders immediately drops to essentially zero; its line cannot easily be seen because it overlaps with the x-axis. Averaged across all parameter combinations the probability of punishing free riders evolves to a higher level in the original simulation set (M = 0.079, SD = 0.059) than in the no-memory replication (M = 0.014, SD = 0.022), t(11998) = 80.054, p<.001, d = 1.46. Given that punishment is effectively nil, there is also little default tendency to cooperate; it hovers around 2–3%. We suspect that this low value represents mutation and drift, not positive selection for default cooperation.

This is not an isolated example. As graphed in Fig 2, in the no memory conditions where inducement is impossible, agents almost always evolve to be completely unwilling to punish—the probability of punishment evolves to be zero. This is true for both mixing and fixed groups ecologies; the lines graphing these two cannot be distinguished because they sit on top of each other at 0%. The lone exception is when the benefits of cooperation are 1 and are therefore identical to the cost of cooperation. In this case, agents should, in the absence of punishment, be indifferent between cooperating and free riding. This may have relaxed selection against punishment, allowing it to drift in to a very small degree. This exception is not general because here free riding is not the only payoff maximizing strategy. In the general case—the bulk of Fig 2—punishment is strongly selected against when cooperation cannot be induced.

### Does a fixed groups ecology underestimate the evolution of punishment?

Yes. This was tested comparing a mixing ecology (agents interacted in multiple, partially overlapping groups throughout their lives) with a fixed groups ecology (agents interacted in a single, fixed group their entire lives). As predicted, agents in the mixing ecology evolved significantly higher levels of willingness to punish than did agents in the fixed groups ecology F(1,5700) = 2040.37, p<.001, η^2^ = 0.02, see Fig 2. Further, this effect was magnified by the gains at stake through recruited labor, such that the manipulation of ecology interacted with both within-round benefits (F(4,5700) = 341.77, p<.001, η^2^ = 0.02), and the number of expected encounters (F(5,5700) = 358.21, p<.001, η^2^ = 0.02). Aggregating across all of the variance components containing the ecology term, ecology influenced approximately 11% of the variance in the evolution of willingness to punish (Table 1).

### Can punishment and cooperation evolve in large groups?

In most models of punishment, being a punisher is all or none: You punish every free rider every time and so the costs of punishment scale with group size. But the benefits of punishment do not scale with group size: Cooperation can be induced by just one punisher and there is no marginal benefit of adding another. With these assumptions, punishment is increasingly selected against as group size gets larger. This has led some researchers to assume that punishment cannot evolve in larger groups without additional cultural mechanisms [20]. Our results challenge this assumption. By using probabilistic punishment, the costs of punishment could be spread throughout the group. As Fig 1 shows, this allows punishment to evolve even in sizable groups. Although willingness to punish does drop as group size increases (F(4,5700) = 6072.06, p<.001, η^2^ = 0.28), it nonetheless evolves high enough to induce a near 95% cooperation rate (Fig 3). In large groups of 25, an average probability of punishment of 10% implies that there is a 90% chance that at least one group member will punish a free rider. Even rare punishment can induce much cooperation.

## Discussion

The results of these simulations are clear: Under minimal yet realistic assumptions, the motivation to punish defections in group cooperation can readily evolve and can stabilize the evolution of motivations to cooperate by default—that is, without being induced to do so by the imminent threat of being punished. Because agents can be willing to cooperate but be unwilling to punish free riders, second order free riders can exist in these populations. Yet they pose no barrier to the evolution of cooperation in groups, because punishers still accrue more benefit from their punishments than do second-order free riders. It is of interest to compare the level of punitiveness that evolved in these simulations to the levels observed in humans. In both cases, moderate levels of punitiveness are observed. Yet, this moderate to low level of punitive tendency—e.g., being motivated to punish defection around 10–40% of the time—was sufficient to elicit extremely high levels of cooperation: on average, agents cooperated between 65%- 95% of the time in the final round of their generation.

Further, it is relevant to point out that punitive tendencies were not identical across meaningful changes in the ecology. For example, punitive tendencies were dramatically lower in ecologies where interactions tended to be brief and cooperation generated little benefit. Secondly, the evolution of punishment was constrained by the level of default cooperation that emerged—the more cooperation, the smaller the residual selection for greater punishment. Third, the potential in the mixing ecologies to recruit labor by expressing a punitive tendency beyond the first round of a fixed group also resulted in greater selection for punishment. These results suggest that, in principle, selection can favor psychological adaptations that are sensitive to these variables (e.g., average potential productivity of group actions, average number of repeated encounters) as well as others, using them to calibrate a person’s punitive disposition. A brain that evolved to calibrate its strategy to factors like these could produce the kind of individual and “cultural” (i.e., geographic or group-based) differences in observed rates of punishment [46] that are often attributed to the work of inherited cultural norms [47]. More generally, the existence of these selection pressures suggests that there should be an evolved, specialized psychology for managing group cooperation and free riders [12,2,48].

Finally, these results join a growing array of evidence that the fundamental components of human social nature—our tendencies to trust and cooperate even with anonymous strangers, our concern for the treatment of others, and our occasional motivation to punish others for their bad behavior—represent an interlocking set of adaptations for deriving benefits from small-scale sociality [27,28,49–52]. Humans interact and cooperate in groups, and occasionally punish those who act poorly. This is but a sliver of our rich group psychology. But group psychology need not have evolved through group selection [53]. These results suggest one plausible alternative.

## Supporting Information

S1 Dataset Simulation Data. Dataset analyzed for GLM analyses. These data represent the average values of the final 500 generations of each simulation run.
(SAV)
